# From spawn to survival: decoding the hydraulic conditions for successful silver carp egg incubation

**DOI:** 10.1371/journal.pone.0320798

**Published:** 2025-04-22

**Authors:** Xian-bing Zhang, Jia-fei Wang, Geng Li, Yu-peng Hu, Wei Yang, Wen-jie Li, Shan Yu

**Affiliations:** 1 Chongqing Jiaotong University, Chongqing, China; 2 National Engineering Research Center for Inland Waterway Regulation, Chongqing, China; 3 Chongqing Key Laboratory of Ecological Waterway, Chongqing, China; 4 Changjiang Chongqing Waterway Engineering Bureau, Chongqing, China; Central University of South Bihar, INDIA

## Abstract

Natural rivers exhibit complex and dynamic flow conditions that significantly influence the survival and development of semi-buoyant fish eggs. This study investigated the effects of flow velocities and turbulence on silver carp eggs (*Hypophthalmichthys molitrix*) during their development. Laboratory experiments conducted in an annular flume revealed that moderate flow conditions (0.5 m/s) yielded optimal hatching rates, while excessive velocities (1.1 m/s) led to complete mortality at the Late Blastula stage. Mild turbulence facilitated egg incubation, whereas intense turbulence reduced hatching success and increased larvae deformation rates. These findings revealed distinct relationship between hydrodynamic conditions and embryonic development, indicating that optimal spawning conditions differ from those required for successful hatching. These results provide fundamental insights for evaluating suitable hydraulic conditions in river habitats and assessing the potential impacts of hydraulic structures on fish populations. The study contributes valuable knowledge to river ecosystem management, semi-buoyant fish species conservation, and fish-friendly hydraulic structure design.

## 1. Introduction

The survival of semi-buoyant fish eggs, including silver carp (*Hypophthalmichthys molitrix*) and grass carp (*Ctenopharyngodon idella*), plays a vital role in maintaining healthy fish populations [1,2]. These eggs are especially susceptible to hydrodynamic conditions during their development in riverine environments [3,4]. After fertilization and subsequent water hardening, which creates a protective perivitelline space [5,6], the eggs must remain suspended in water currents to survive. They can drift vast distances (hundreds of kilometers) before reaching suitable nursery habitats [7], which makes them susceptible to varying flow velocities and turbulence intensities [3,8]. While optimal spawning flow velocities for silver carp have been identified (1.4–1.6 m/s), the specific effects of flow velocities and turbulence intensities on egg incubation and hatching remain poorly understood [4,9].

Although turbulence is essential for maintaining egg suspension, excessive turbulence, especially when combined with high flow velocities, can negatively impact embryonic development [5,6,10]. This results from physical stress such as continuous fluctuation and rotation, shear forces, and abrasion [1,9,10,11]. Higher flow velocities have been shown to negatively correlate with hatching rates and larval survival [1,9,10–12]. The physical stresses associated with high velocities, including constant fluctuations, shear forces, and abrasion, likely contribute to reduced hatching success. This underscores the complex interplay between hydrodynamics and early life stages in riverine ecosystems and highlights the need for further research to inform effective conservation strategies.

The embryonic development of silver carp egg, from fertilization to hatching, is a complex process encompassing over 30 distinct ontogenetic stages [13,14]. Each stage exhibits unique morphological and physiological characteristics, potentially susceptible to hydrodynamic stress. To facilitate systematic investigation and optimize observational protocols, researchers have stratified this intricate developmental continuum into seven key stages [1,2]. These stages include: I—1-cell, characterized by perivitelline space formation; II—Cleavage, marked by rapid cell divisions; III—Blastula, signified by yolk syncytial layer emergence; IV—Gastrula, involving the establishment of the trilaminar germ disc (ectoderm, mesoderm, and endoderm); V—Neurula, distinguished by neural plate and neural fold development; VI—Organ differentiation, differentiation of various organ systems; VII—and hatching, detectable heartbeat and tail movements. This simplified ontogenetic framework enables a more focused analysis of the correlation between diverse hydrodynamic conditions and their impact on hatching success rates and larval viability across these critical developmental milestones.

To gain deeper insights into the hydrodynamic conditions that significantly impacts on the embryonic development and early life stage survival of semi-buoyant fish eggs, we conducted an experimental study using silver carp eggs as the representative species. This investigation aimed to elucidate the effects of velocity variation and turbulence on the development and survival potential of silver carp eggs and hatched larvae. The experimental protocol improved upon previous research by precise hydrodynamic measurements, monitoring the biological development of eggs and larvae during incubation, and assessing malformation and mortality induced by flow conditions. Through this systematic examination of key developmental stages and their interaction with hydrodynamic factors, researchers seek to enhance our knowledge of the environmental conditions crucial for early survival of silver carp. Such insights are invaluable for conservation efforts and the management of aquatic ecosystems where these species play a significant role.

## 2. Materials and Methods

### 2.1 Collection of sliver carp eggs

The Animal Care and Use Committee of Chongqing Jiaotong University granted us permission to possess silver carp (*Hypophthalmichthys molitrix*) eggs and larvae in a laboratory setting for research purposes only (Permit No. Wang-20220325-002). The silver carp eggs were obtained from Yongchuan Shuihua Fishery Association, a fishery farm located in Chongqing, (105.9°E, 29.263°N), from May to July in 2022 and 2023.

### 2.2 Design of annular flume

**Fig 1:**
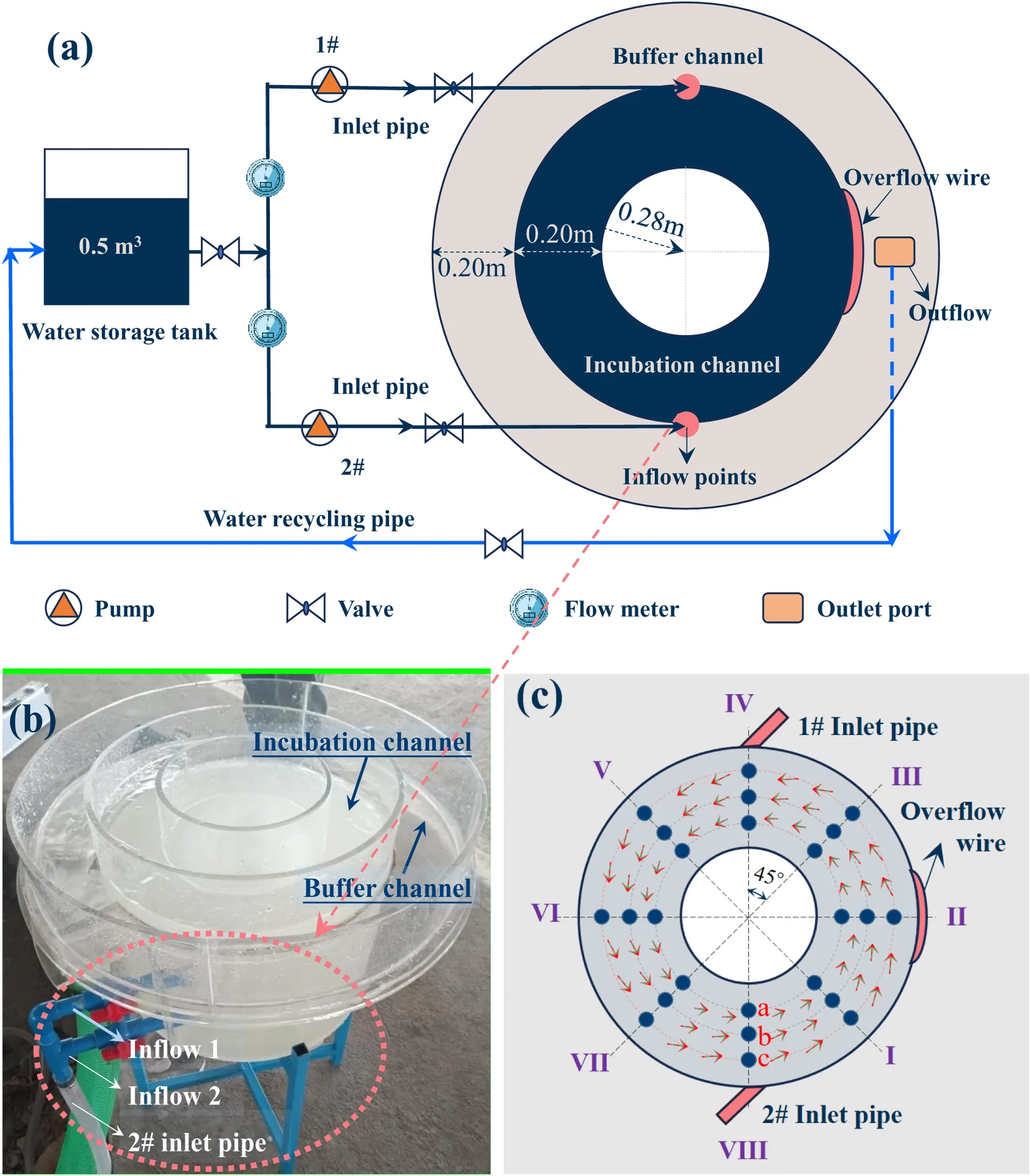
Annular flume (AF) for silver carp egg incubation. (a) Schematic diagram of the AF setup. (b) Photograph of the assembled AF apparatus. (c) Distribution of flow velocities measurement points within the AF.

An annular flume (AF) was designed and constructed for this study to investigate silver carp egg development under controlled flow conditions. The AF, depicted in Fig 1, comprises three main components aligned at the top: an incubation channel, a buffer channel, and an overflowing weir.

The incubation channel, serving as the primary experimental area, measures 0.20 m in width and 0.40 m in height, with a working water depth of 0.30 m. Adjacent to this, the buffer channel has the same width but reduced height of 0.20 m. This design ensures stable flow transition between channels.

Flow control was achieved through two inlet pipes, each followed by two inflows. The angles of these inflows were adjusted to either 45° or 135°, allowing precise regulation of flow conditions in the incubation channel by modifying inlet pipe flow rates (S1 Fig).

A low barrier, or weir (0.35 m wide and 0.10 m high), spans the wall between the incubation channel and the buffer channel to control water level. Excess water flows over this weir, while a fine mesh (1 mm square grid, constructed from hand-woven, unbleached, multi-strand cotton yarn) covering the weir prevents egg loss.

The annular flume dimensions were chosen based on a balance between creating a controlled, measurable flow environment and mimicking aspects of natural river flow conditions relevant to silver carp spawning. While no specific literature exists recommending these exact dimensions for a flume study of this nature, our design was informed by studies on fish egg and larval transport, which emphasize the importance of controlled flow conditions for studying the effects of turbulence. The 0.20 m width of the incubation channel was a compromise to allow for controlled turbulence generation while still providing sufficient space for egg movement. Silver carp are known to spawn in fast flowing riverine environments, and our flume design aimed to replicate these flow characteristics, specifically the turbulence intensity, while allowing for precise control and measurement of flow parameters.

### 2.3 Characterization of flow conditions

(1) Measurement of flow velocities

A comprehensive three-dimensional flow velocities measurement was conducted in AF using an Acoustic Doppler Velocimeter (ADV, Vector 300m, Nortek Engineering AS Vector. Norway; detailed specifications available in S1 Text). The experimental configuration consisted of 72 systematically distributed measurement points distributed across the flume. As illustrated in Fig 1 (c), these points were organized into 8 cross-sections along the flume length. On each cross-section, 3 vertical perpendiculars were set with an interval of 0.05 m. For every vertical perpendicular, data were collected at three relative heights: 0.2, 0.6, and 0.8 times the water depth. This systematic arrangement ensured a thorough capture of the flow dynamics throughout the AF, yielding a comprehensive dataset for analysis.

The average velocity on each perpendicular line (*U*_i_) was calculated according to equation (1).


$$\begin{array}{c} {\text{U}}_{\text{i}}\text{=}\frac{\text{1}}{\text{3}}({\text{U}}_{\text{0.2h}}+{\text{U}}_{\text{0.6h}}+{\text{U}}_{\text{0.8h}}) \end{array}$$ (1)

Where *h* was the water depth in the incubation channel. *U*_0.2*h*_, *U*_0.6*h*_, and *U*_0.8*h*_ represent the velocities at 0.2, 0.6, and 0.8 times the water depth.

The average velocity on each cross-section *U*_si_ was determined using equation (2).


$$\begin{array}{c} {\text{U}}_{\text{si}}\text{=}\frac{\text{1}}{\text{3}}({\text{U}}_{\text{ia}}+{\text{U}}_{\text{ib}}+{\text{U}}_{\text{ic}}) \end{array}$$ (2)

Here, *U*_ia_, *U*_ib_, and *U*_ic_ represent the average velocity of a, b, and c perpendicular lines on the same cross-section.

The determination of average flow velocity in the egg incubation channel was accomplished through a calculation based on two key parameters: the flow volume measured by the flow meter with accuracy of 0.1 L/s and the cross-sectional area (height×width = 0.3× 0.2, m^2^) of the channel. This method provides a reliable estimate of the overall water movement within the experimental setup. However, it is crucial to note that this calculated velocity value is not constant throughout the incubation period. The water surface in the channel is subject to natural fluctuations, which in turn cause variations in the flow velocity.

(2) Calculation of turbulent intensity and kinetic energy

The turbulent intensity and kinetic energy of each measuring point were measured and calculated by the ADV system.

The turbulent intensity in the *x*-direction (streamwise) is represented by $\sigma_{x}$.


$$\begin{array}{c} \sigma_{x}=\sqrt{\overset{―}{u_{x}^{\prime_{2}}}}=\sqrt{\frac{\sum {\left(u_{x}-\overset{―}{u_{x}}\right)}^{2}}{n}} \end{array}$$ (3)

The turbulent intensity in the *y*-direction (cross stream) is $\sigma_{\text{y}}$.


$$\begin{array}{c} \sigma_{\text{y}}\text{=}\sqrt{\overset{―}{{\text{u}}_{\text{y}}^{\text{'2}}}}\text{=}\sqrt{\frac{\sum {\left({\text{u}}_{\text{y}}\text{-}\overset{―}{{\text{u}}_{\text{y}}}\right)}^{\text{2}}}{\text{n}}} \end{array}$$ (4)

The turbulent intensity in the *z-*direction (vertical) is $\sigma_{\text{z}}$.


$$\begin{array}{c} \sigma_{\text{z}}\text{=}\sqrt{\overset{―}{{\text{u}}_{\text{z}}^{\text{'2}}}}\text{=}\sqrt{\frac{\sum {\left({\text{u}}_{\text{z}}\text{-}\overset{―}{{\text{u}}_{\text{z}}}\right)}^{\text{2}}}{\text{n}}} \end{array}$$ (5)

The turbulent energy is calculated by Eq. (6).


$$\begin{array}{c} \text{k=}\frac{\text{1}}{\text{2}}(\sigma_{\text{x}}^{\text{2}}\text{+}\sigma_{\text{y}}^{\text{2}}\text{+}\sigma_{\text{z}}^{\text{2}})\text{=}\frac{\text{1}}{\text{2}}(\overset{―}{{\text{u}}_{\text{x}}^{\text{'2}}}\text{+}\overset{―}{{\text{u}}_{\text{y}}^{\text{'2}}}\text{+}\overset{―}{{\text{u}}_{\text{z}}^{\text{'2}}}) \end{array}$$ (6)

Here, $u_{x}^{\prime }$, $u_{y}^{\prime }$, and $u_{z}^{\prime }$ are the pulsating velocity (m/s) in the *x*, *y*, and *z* directions, respectively. Additionally, *u*_*x*_, *u*_*y*_, *u*_*z*_ represent the instantaneous flow velocity (m/s) in the *x*, *y*, and *z* directions, respectively. ${\overset{―}{u}}_{x}$, ${\overset{―}{u}}_{y}$, and ${\overset{―}{u}}_{z}$ represents the average flow velocities (m/s) in the respective directions. While *n* represents the number of instantaneous flow velocities. The turbulent energy *k* value (m^2^/s^2^) characterizes the turbulent level in the annular flume.

(3) 2-D cloud map of flow velocities and turbulent intensities

To enhance the visualization and understanding of the flow field within the incubation channel, we employed Surfer V.15.0 software to generate 2-D cloud maps depicting flow velocities and turbulence intensities. The spatial interpolation of our data was accomplished using Shepard’s method, a sophisticated three-dimensional interpolation technique integrated into the Surfer software package. This approach involved a two-step process: first, we plotted the in-situ average flow velocities derived from Eq. (2) and then estimated velocities in unmeasured areas through above interpolation method. For the 2-D cloud map illustrating turbulent intensity, we exclusively utilized data from Eq. (3), which specifically represented turbulence intensity in the x-direction. This focus on the x-direction was justified by the observation that turbulence intensity differences in the y-direction and z-direction were not statistically significant.

### 2.4 Selection of experimental parameters

High-speed turbulent streams are commonly found in mountain rivers such as the upper reaches of the Yangtze River [15–18]. These flows are crucial for the spawning of certain fish species, including the four major Chinese carps, which require a flow velocity of approximately 1.1 m/s to release their eggs [9]. Additionally, a specific flow velocity is needed to keep semi-buoyant fish eggs suspended and alive [10]. Therefore, flow velocities ranging from 0.3–1.1 m/s were utilized in the current study.

This comprehensive study investigated the effects of both flow velocities and turbulence intensities on silver carp egg incubation through two interconnected experimental series. This first series comprised six experiments examining flow velocity impacts. Five primary experiments (L1-L5, Table 1) utilized distinct average flow velocities (0.3, 0.5, 0.7, 0.9, and 1.1 m/s) in the incubation channel, while a control experiment (L0) employed a separate zero-velocity incubator (ZET-80, ZISS Tumbler, Foshan, Guangdong, China). All experiments maintained a 0.3 m average water depth, with temperature continuously monitored in the buffer channel using a thermometer (model 1621A, manufactured by Hefei Zhice Electronic Co., Ltd., Hefei, China).

**Table 1:** Flow parameters in the study of flow velocity influence the incubation and survival of silver carp egg and hatched larvae.

Number	Mean flow velocity (m/s)	Water depth, *h* (m)	Hydraulic radius, *R* (m)	*ν* ×10^–6^ (m^2^/s)	Reynolds number, Re	Froude number, Fr	Flow inlet method
L0	0.0	–	–	–	–	–	In a separate incubator
L1	0.3	0.3	0.043	0.8545	15096.55	0.175	1# inlet pipe + 2 inflows
L2	0.5	0.3	0.043	0.8545	25160.91	0.292	1# inlet pipe + 2 inflows
L3	0.7	0.3	0.043	0.8545	35225.28	0.408	1# inlet pipe + 2 inflows
L4	0.9	0.3	0.043	0.8545	45289.64	0.525	1# inlet pipe + 2 inflows
L5	1.1	0.3	0.043	0.8545	55354.01	0.642	1# inlet pipe + the lower inflow

The second experimental series focused on turbulent intensity effects, consisting of four primary experiments (W1–W4, Table 2) and a control (W0). The primary experiments maintained a consistent 0.7 m/s mean flow velocity while varying turbulent intensities (0.0968, 0.1044, 0.1297, and 0.1439 m/s) in x-direction. As before, a separate zero-velocity incubator serves as the control (W0).

**Table 2:** Turbulent flow parameters for silver carp egg and larvae survival study.

Number	Mean flow velocity (m/s)	Average turbulent intensity in *x* direction (m/s)	Average kinetic energy (m^2^/s^2^)	Flow inlet method
W0	–	–	–	In a separate incubator
W1	0.7	0.0968	0.0119	2# inlet pipe + 2 inflows
W2	0.7	0.1044	0.0166	1# inlet pipe + 2 inflows
W3	0.7	0.1297	0.0249	1# inlet pipe + upper inflows
W4	0.7	0.1439	0.0281	2# inlet pipe + lower inflows

### 2.5 Experimental procedure

All experiments were conducted at the fishery farm. Prior to each experiment, flow conditions in the incubation channel were carefully adjusted to achieve the desired flow velocities and turbulence intensities, ensuring a stable environment. For every experimental run, 60 newly fertilized silver carp eggs at the 1-cell stage (approximately zero post-fertilization age) were carefully selected and transferred from the fishpond to the prepared channel. Ten of these eggs were randomly selected for observation of morphology and health status. These selected eggs were observed and photographed for a few seconds before being returned to the experimental channel. To ensure statistical robustness and reproducibility, each experiment was replicated three times, resulting in a comprehensive study involving 1080 fertilized silver carp eggs. Throughout the incubation period, water quality was rigorously monitored to maintain dissolved oxygen levels at a minimum of 7 mg/L, thereby providing optimal conditions for egg development.

Throughout the experiment, meticulous observation of silver carp eggs development was conducted at regular intervals. During the initial stages, eggs were examined every 30–60 minutes using a high-resolution portable microscope (SHOCREX DM9, 1000X magnification, Shenzhen Shu’an Technology Development Co., Ltd., Shenzhen, China). Once the organ differentiation stage was reached, observation intervals were extended every 2–3 hours until hatching. The morphology and health status of silver carp eggs and larvae exposed to high-speed stream and turbulence were observed.

Particular attention was paid to the morphology and health status of eggs and larvae exposed to high-speed streams and intense turbulence. The water temperature was strictly controlled at 28 ± 0.5°C and continuously monitored throughout the experiment. The onset of each developmental stage was defined as the point when over 50% of the fertilized eggs reached that specific stage. The total incubation duration, which varied between 21- and 23-hours post-fertilization depending on specific conditions, was recorded along with egg mortality rates at each stage.

### 2.6 Calculation of hatching rate and deformity rate

The developmental stages of fish eggs are divided according to Amy E. George’s method [2], the eggs are divided into more than 30 different stages of development. The hatching rate (%) of silver carp eggs and the deformity rate (%) of hatched larvae were calculated using the following equations.


$$\begin{array}{c} Z_{1}\;=\;\frac{Y_{1}}{Y}\;\times \;100\% \end{array}$$ (7)

$$\begin{array}{c} Z_{1}\;=\;\frac{Y_{2}}{Y}\;\times \;100\% \end{array}$$ (8)

Here, *Z*_1_ is the hatching rate, *Z*_2_ is the deformity rate, *Y*_1_ is the hatched larvae, *Y*_2_ is the deformed fish larvae, and *Y* represents the initial number of silver carp eggs.

## 3. Results

### 3.1 Spatial distribution of flow in the incubation channel

**Fig 2:**
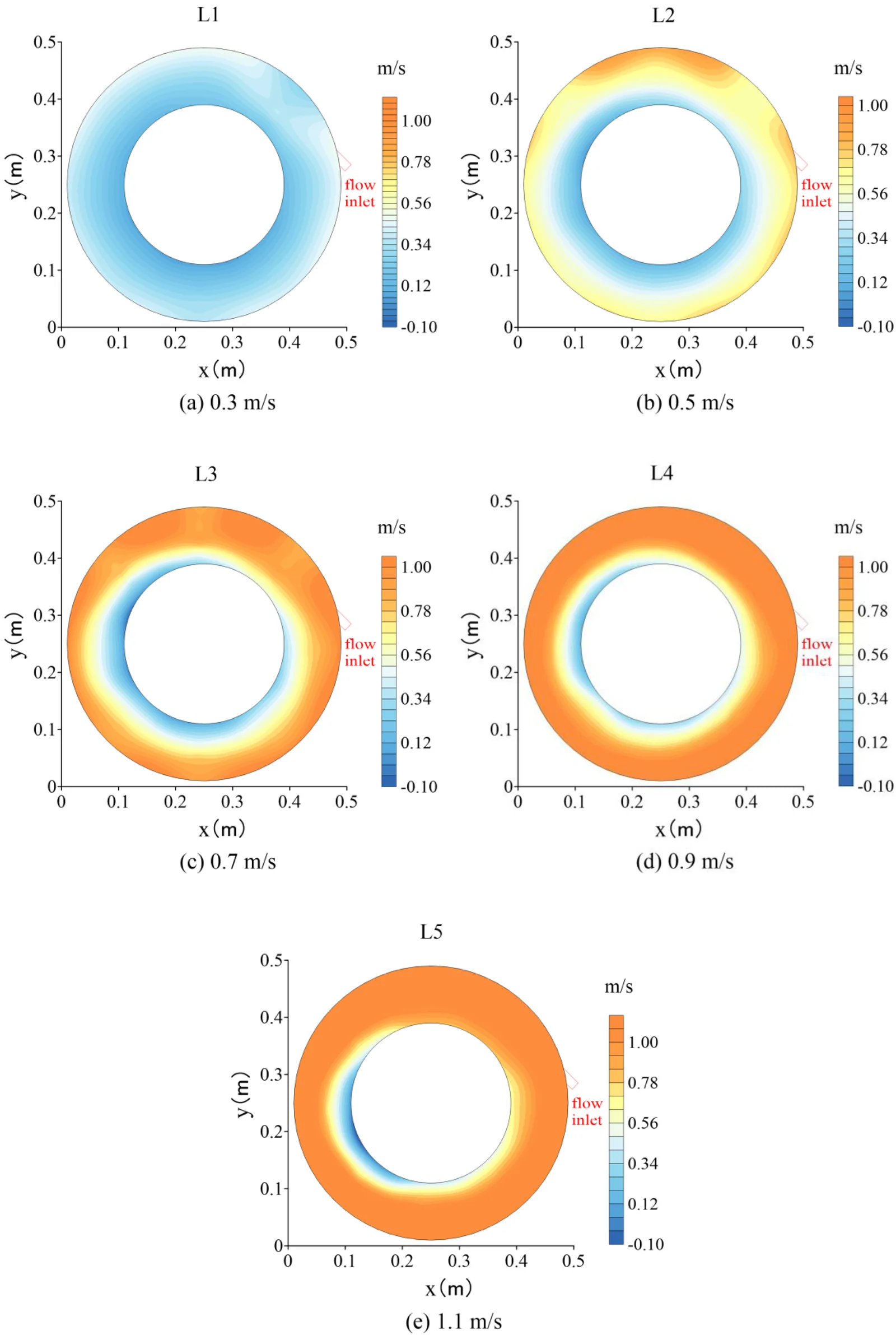
Spatial distribution of flow velocities in the incubation channel under varying operational flow conditions.

**Fig 3:**
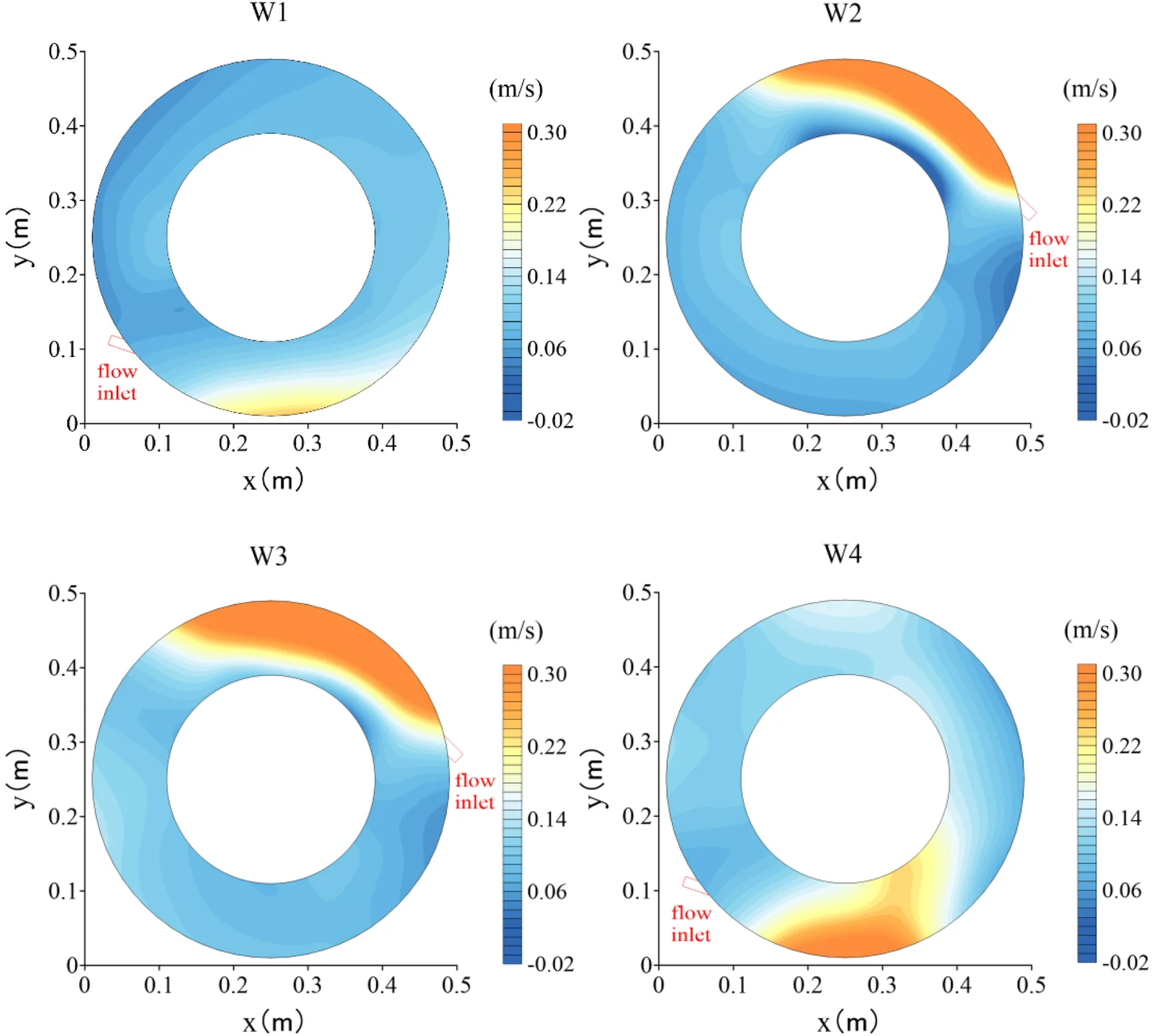
Spatial distribution of turbulence intensities in the incubation channel under varying operating conditions at constant mean flow velocity (0.7 m/s).

Flow characteristics in the incubation channel were visualized using 2-D cloud maps for velocity distribution (Fig 2) and calculated turbulent intensities in the *x* direction (W1–W4, Fig 3). Lower average flow velocity was associated with more uniform distribution of flow velocities and stable conditions. The velocity pattern consistently demonstrated lower velocities near the channel’s inner wall, increasing towards the periphery, with higher velocities near the inlet. This distribution pattern remained consistent across different overall flow velocities. Notably, turbulence intensity in the y-direction exhibited similar and comparable values throughout the channel.

The spatial distribution patterns of turbulent intensities within the incubation channel showed the highest and most variable near the flow inlet. while other areas exhibited relatively stable and weak turbulence intensities. The distribution pattern of turbulent energy in the incubation channel mirrored that of turbulent intensity under the same experimental conditions (S2 Fig), indicating a correlation between higher turbulence intensity and greater turbulent energy. In the flow inlet region, the maximum turbulent kinetic energy (*k*_max_) reached approximately 0.1 m^2^/s^2^. This localized area of intense turbulence differed significantly from the rest of the system. Other areas exhibited markedly different characteristics, with turbulence intensities remaining relatively stable and weak. This spatial heterogeneity in turbulent intensity suggests a complex flow structure, with the inlet region acting as a hotspot for turbulent energy dissipation. Thus, both the maximum and mean turbulence intensity were adopted to evaluate the intensities across different experimental conditions.

### 3.2 Influence of flow velocities and turbulence intensities on the hatching performance of silver carp eggs

**Fig 4:**
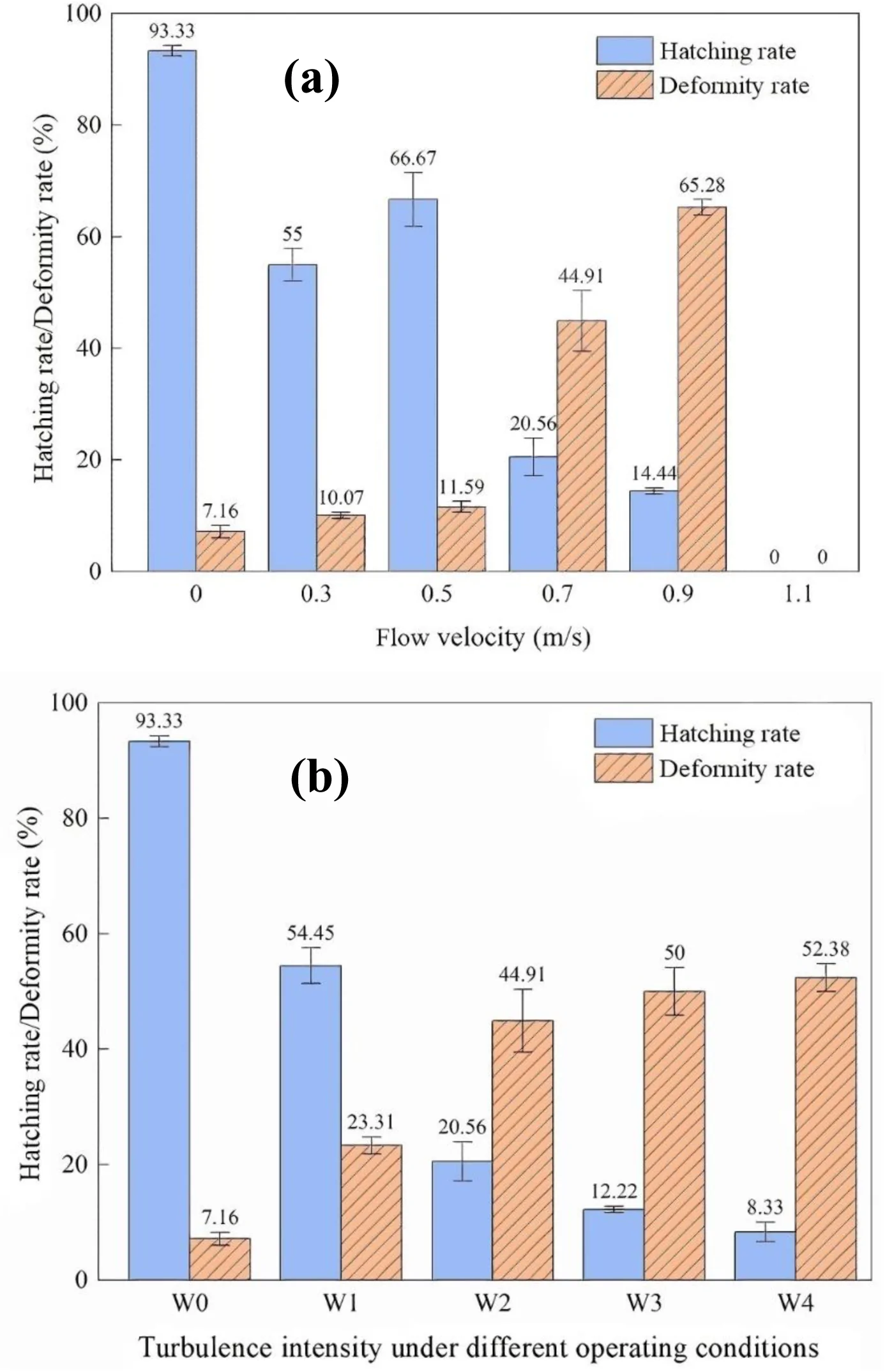
Impacts of mean flow velocities (a) and turbulence intensities on the hatching performance larvae deformity rates of silver carp.

The study maintained average flow velocities in the incubation channel between 0.3–1.1 m/s to investigate the effects of flow velocity on the incubation of silver carp eggs. By examining the effect of flow turbulence on the hatching and survival of silver carp eggs and larvae at a consistent flow velocity (0.7 m/s) with varying turbulence intensity, it was found that the hatching rates were higher in stagnant water compared to moving water (Fig 4(a)). Hatching rates increased to around 66% at 0.5 m/s, but then sharply dropped to 14% at 0.9 m/s. Notably, eggs did not hatch at 1.1 m/s, with all embryos dying before reaching the Late-Gastrula stage. As turbulence intensity and energy increased, hatching rates decreased and deformity rates rose (Fig 4(b)), suggesting that fish eggs thrive in environments with lower flow fluctuations and more stable flow conditions.

Larval health was significantly affected by flow conditions. Deformity rates increased from 7% in the control group to 10–12% at flow velocities of 0.3–0.5 m/s, and exceeded 40% at velocities above 0.7 m/s. Hatching rates dropped from 93% in the control to below 20% when turbulence intensities surpassed 1.0 m/s. These results underscore the critical impact of flow turbulence intensity on both hatching success and larval development.

**Fig 5:**
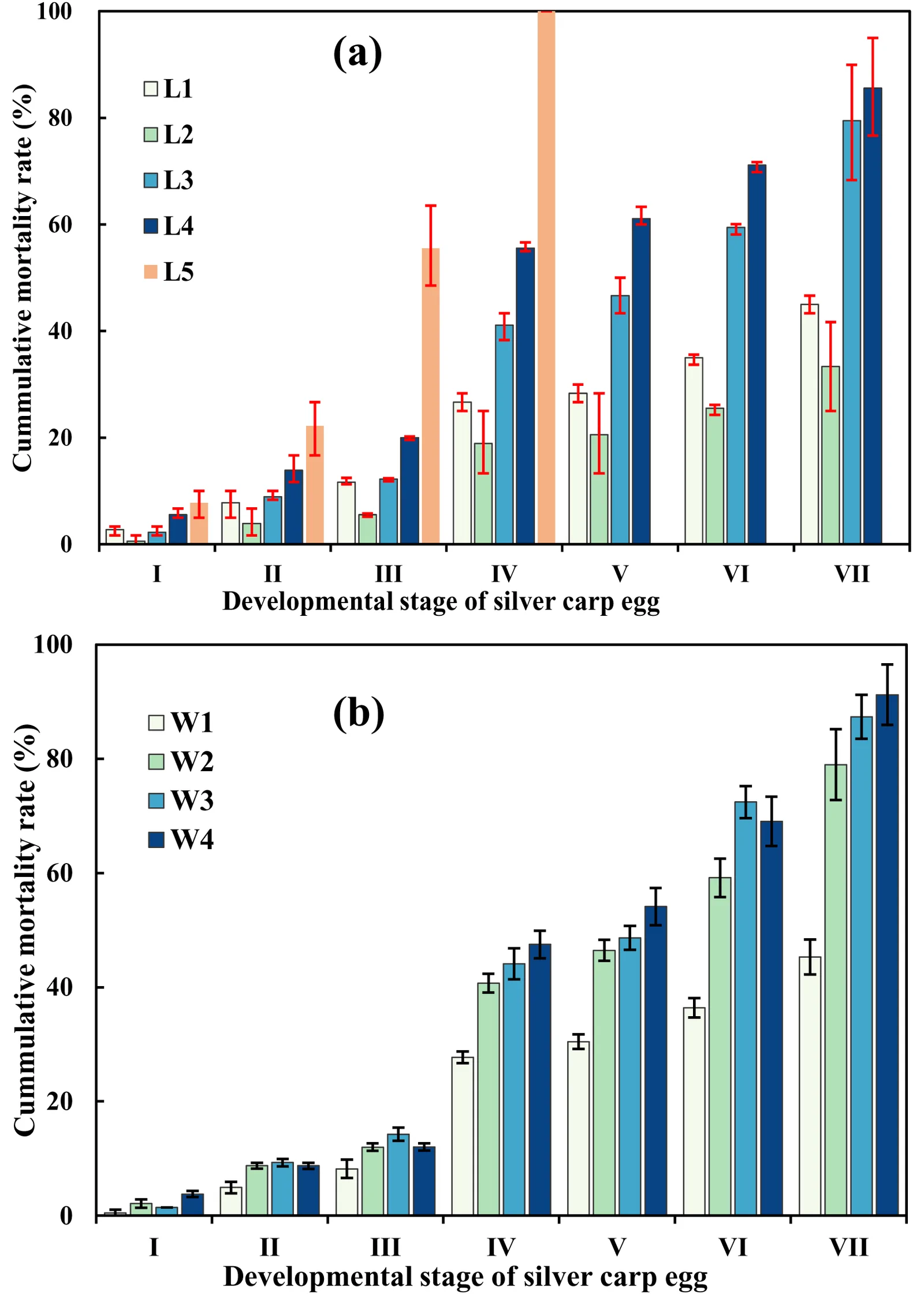
Cumulative mortality of silver carp eggs undergoing various flow velocities ((a), L1–L5 with corresponding average flow velocity of 0.3, 0.5, 0.7, 0.9, 1.1 m/s) and turbulence intensities ((b), W1–W4 represent operation turbulence intensities of 0.0968, 0.1044, 0.1297, 0.1439  m^2^/s^2^ with same average velocity of 0.7 m/s) at different development stage.

Egg mortality varied across developmental stages under different flow conditions. In the 0.0–1.1 m/s velocity range, the Mid-Gastrula stage (VI) showed the highest vulnerability, with average mortality rates of 15%, 13%, 29%, 36%, and 44% under conditions L1-L5, respectively. Subsequent stages in order of susceptibility were hatching, organ differentiation, cell cleavage, blastula, neurula, and 1-cell (Fig 5). For turbulence intensity, the blastula stage was most affected, with average mortality rates of 20%, 29%, 30%, and 35% under conditions W1-W4, followed by Hatching, Lens formation, 128-cell, Mid-Gastrula, Neurula, and 1-cell stages.

### 3.3 Abnormal development of silver carp eggs and larvae

**Fig 6:**
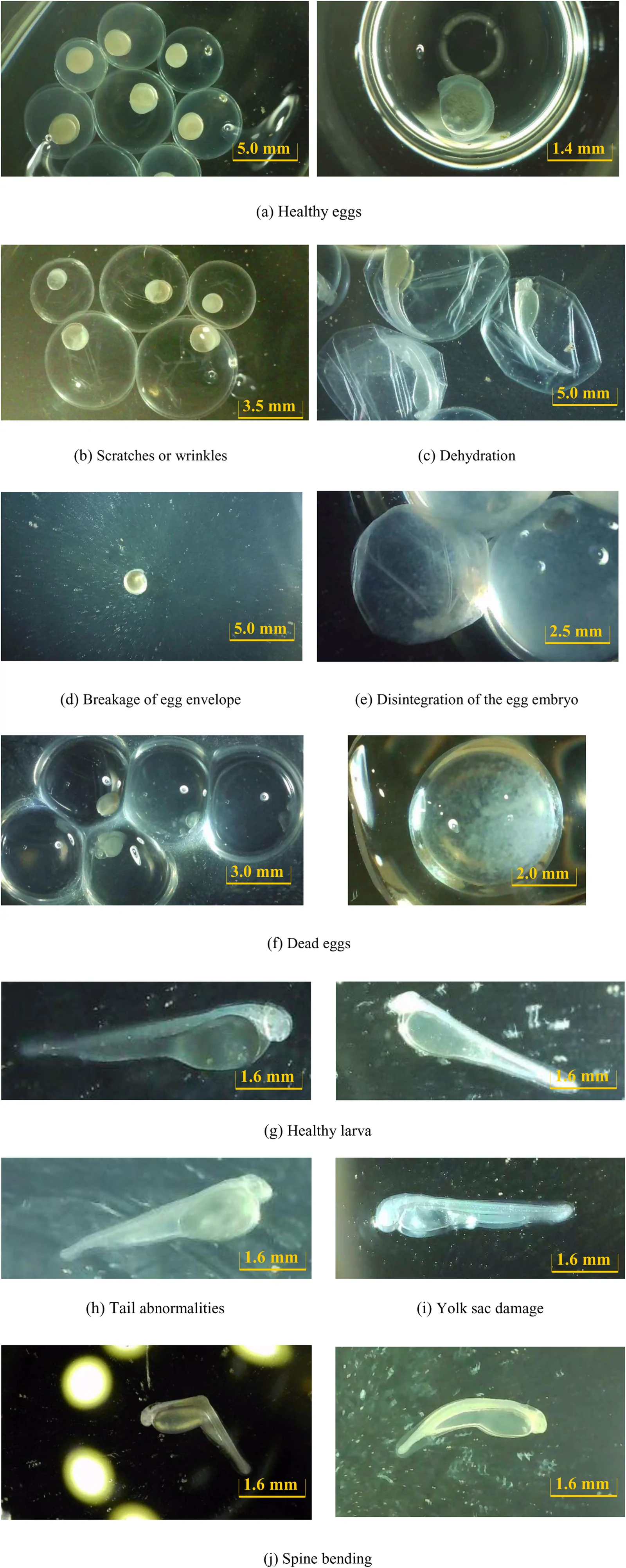
Abnormal development of the silver carp eggs and larvae when suffering continuous high-speed streams and turbulence.

Silver carp eggs undergo distinct developmental stages from fertilization to hatching, each characterized by specific morphology [2]. Our field investigation team’s extensive observation of early-stage fish resources in the upper Yangtze River revealed that exposure to high-speed streams and turbulent flows can result in abnormal changes in the morphology of semi-buoyant fish eggs and larvae. While healthy eggs typically appear plump and smooth, abnormal development in silver carp eggs may present as surface scratches, wrinkles, and cracks, along with varying degrees of dehydration, envelope breakage, and embryo disintegration (Fig 6(a–f)). These types of damage can lead to deformities in hatched larvae, including spine curvature, tail irregularities, and yolk sac damage (Fig. 6(g–j)). These abnormalities ultimately contribute to fish larvae mortality. So, the morphology of fish eggs and larvae was carefully checked throughout all the experiments.

When incubation flow velocities were 0.3 m/s and 0.5 m/s, fish eggs exhibited spherical morphology with intact surfaces (Fig 6(a)). As incubation progressed, more and deeper cracks emerged on the surface of the egg envelope (Fig 6(b)). These cracks led to a gradual loss of liquid between the embryo and the envelope, resulting in the eggs becoming slightly dehydrated and then semi-dehydrated. Nevertheless, partially dehydrated fish eggs were still able to develop into organ formation or hatching stage with fully developed physiological functions when compared to the control group.

When the incubation velocities exceeded 0.7 m/s, the damage to the egg envelope worsened, potentially resulting in complete dehydration of the fish eggs. Additionally, higher flow velocities led to accelerated water loss in the eggs. Some eggs exposed to flow velocities of 0.7 m/s and 0.9 m/s experienced complete dehydration before reaching the organ differentiation stage (Fig 6(c)). Furthermore, prior to hatching, most egg envelopes were completely ruptured when the running flow velocity was 1.1 m/s (Fig 6(d)). In the absence of protective envelopes, fish eggs are susceptible to rapid death from collision shock, impeding normal hatching and significantly increasing the mortality rate.

Egg dehydration was observed to accelerate with higher turbulence intensity. Before the onset of organ differentiation, eggs were fully dehydrated when turbulence intensity surpassed 0.1 m/s in the *X* direction. At the hatching stage during these high turbulence intensities, egg envelopes ruptured and stuck to the embryo, impeding hatching and making embryos vulnerable to physical harm. Consequently, the hatching rate significantly decreased when turbulence intensity exceeded 0.1 m/s.

## 4. Discussion

The study found that the hatching rate of silver carp eggs in a laboratory flume significantly decreased when flow velocities exceeded 0.7 m/s with all eggs dying by the blastula stage at an average flow rate of 1.1 m/s. These results indicate that slow flows with low turbulence intensity are advantageous for silver carp eggs incubation if turbulence is sufficient to eggs in suspension. However, optimizing flow velocity within a suitable range could enhance hatching performance. It is likely that an optimal flow velocity facilitates material exchange, such as dissolved oxygen, between eggs and their environment, thereby improving the hatching success. These findings can help evaluate hydraulic conditions for the drifting and incubation of semi-buoyant fish eggs, considering specific river habitat characteristics. In natural rivers, maintaining high flow velocity is crucial for spawning stimulation [9]. Nevertheless, high flow velocities can lead to increased egg mortality [1,19,6]. This means the flow conditions may be suitable for spawning, but they may not be conducive to egg hatching. Therefore, when implementing ecological management strategies to promote fish reproduction, it is important to consider not only the flow velocity necessary for spawning but also the appropriate flow velocity for egg hatching.

Flow turbulence plays a crucial role in the successful hatching of semi-buoyant fish eggs by maintaining their suspension [20,21]. However, our experiments further revealed that excessive turbulence can be detrimental. We observed a 40% decrease in hatching rate and 30% increase in deformity rate as turbulence intensity increased from 0.0968 to 0.1439 m/s at a mean flow velocity of 0.7 m/s with maximum turbulence intensity of approximately 0.3 m/s. Prada et al. examined grass carp egg resilience to turbulence [10]. In their experimental setup, Prada and colleagues utilized a grid-stirred tank to simulate turbulent conditions. They subjected water-hardened grass carp eggs to short-duration turbulence events lasting one minute. Their results demonstrated a clear correlation between turbulence intensity and egg survival rates. Specifically, the survival rates decreased from 90% to 70% when maximum kinetic energy (*k*_max_) increased from 2.0 to 2.7 m^2^/s^2^ in a grid-stirred tank with exposure time at one minute. The researchers proposed a mechanistic explanation for this observation, suggesting that the increased mortality was likely due to the eggs’ exposure to fast-moving suspended sediments, which become more prevalent and energetic under higher turbulence conditions. Notably, the turbulence intensity and kinetic energy in present setup were lower than both Prada et al.’s experiment and those typically found in natural rivers. Nevertheless, our results suggest that the hatching performance and survival potential of drifting fish eggs would be significantly affected in continuous high-speed streams with turbines, even at these lower turbulence levels.

Mechanical shock and embryo disintegration represent the primary mechanisms of egg mortality in high-speed streams. The primary reason is the mechanical shock from turbulence-induced collision with the flume wall in the y-direction (cross stream). Unfortunately, the frequency and magnitude of these embryo collisions were not quantified in this study. Previous research has shown that the impact of sudden and intense collision shock induced by high-speed streams on the hatching of semi-buoyant fish eggs is influenced by collision velocity, substrate of collision, and developmental stage of the eggs [1,10]. Eggs largely survive temporary collisions at 4.4 m/s in free-fall experiments, yet continuous high-speed flows significantly reduce hatching rates. The presence of scratches, wrinkles, cracks on the egg envelopes, and egg envelope breakage serve as clear evidence of collisions (Fig 6(b)–(d)). Once cracks appear, fast-moving eggs dehydrate, hindering their development. Moreover, the rupture of the egg envelope exposes the embryo to harm, leading to egg mortality. Furthermore, silver carp eggs exhibit differential susceptibility to mechanical perturbations across their developmental stages. Yang et al. identified the cell-cleavage stage as the most vulnerable to acute, high-intensity free-fall collision shocks. In contrast, our current flume experiments demonstrated that the blastula stage displayed the highest sensitivity to sustained flow turbulence [1]. These results demonstrate the stage-specific responses of silver carp eggs to diverse forms of mechanical stress, highlighting the complexity of their early developmental physiology.

The other reason for the death of silver carp eggs drifting in high-speed streams can be attributed to the disintegration of the embryos. This disintegration is likely due to prolonged exposure to high-speed turbulent flows, causing the eggs to move and rotate within the water. A similar effect can be observed when vigorously shaking a chicken egg, resulting in a disrupted interior. Based on long-term field observations since 2019 in the upper reaches of the Yangtze River revealed a high incidence of damaged semi-buoyant fish eggs, characterized by compromised embryonic structures. This observation is characteristic of the high-speed turbulent flows prevalent in the area.

During the study, it was found that fish egg mortality was primarily caused by the stalling of embryonic development before reaching the blastula stage. This critical phase made them susceptible to high-speed streams with strong turbulence, leading to higher mortality rates. Throughout the incubation period, silver carp eggs experienced continuous mechanical shock, resulting in frequent mortality during organ differentiation and hatching stages.

This investigation lays a scientific groundwork for examining the quantitative correlation between flow velocity, turbulence, and the hatching of semi-buoyant fish eggs. Additionally, it offers practical implications for implementing strategies to protect the reproduction of semi-buoyant fish eggs using hydraulic characteristics. Moving forward, the experimental flow conditions can be fine-tuned to determine the ideal incubation flow velocity for semi-buoyant fish eggs.

While our current experimental setup has provided valuable insights into the effects of flow conditions on silver carp egg development, we acknowledge certain inherent limitations in experimental design. The relatively narrow flume width, though allowing for precise control of flow parameters, may not fully represent the complex hydraulic conditions found in natural river systems. These findings suggest the need for advanced experimental protocols with broader channels and diversified flow regimes. This would help better simulate natural river conditions while minimizing wall collision effects that may have influenced our current results. Additionally, the integration of advanced imaging and velocity measurement systems would enhance our understanding of egg movement patterns and secondary flow characteristics, particularly in relation to varying channel geometries that more closely mirror natural river morphology. Such advancements would complement our current findings and provide a more comprehensive understanding of the relationship between hydraulic conditions and semi-buoyant fish egg development in natural river systems.

## 5. Conclusion

This study provides valuable insights into the effects of flow velocities and turbulence intensities on silver carp egg incubation and hatching.

The research reveals that moderate flow velocities around 0.5 m/s yield optimal hatching rates, while velocities exceeding 0.7 m/s significantly reduce egg survival and increase larval deformities. Increased turbulence intensity correlates with decreased hatching rates and higher deformity rates, highlighting the importance of stable flow conditions for egg development. Different developmental stages exhibit varying susceptibility to flow conditions, with the gastrula stage being most vulnerable to high velocities and the blastula stage to turbulence.

High-speed streams and turbulence cause physical damage to eggs, including dehydration, envelope breakage, and embryo disintegration, leading to increased mortality [10]. These findings have important implications for river management, emphasizing the need to consider both spawning and hatching requirements when implementing ecological strategies for semi-buoyant fish species.

This research establishes a quantitative foundation for understanding the relationship between hydraulic characteristics and semi-buoyant fish egg development, offering valuable guidance for protecting and managing these species in natural river systems and artificial breeding environments. Future studies should focus on refining experimental conditions to determine optimal incubation flow velocities for semi-buoyant fish eggs, further enhancing our ability to support their reproduction and survival.

## Supporting information

S1 Fig Longitudinal section and dimension annotation of incubation channel and buffer channel, and the inflows setup. (DOCX)

S2 Fig Turbulent Energy Distribution in Incubation Channel at Constant Flow Velocity (0.7 m/s). (DOCX)

S1 File Introduction of ADV. (DOCX)

## References

[1] Yang W, Zhang X-B, Li G, Qin D, Li W, Hu Y. Effects of collision shock on semi-buoyant fish egg hatchings in high-speed streams. Glob Ecol Conserv. 2024;49:e02785. doi: 10.1016/j.gecco.2023.e02785

[2] George AE, Chapman DC. Aspects of embryonic and larval development in bighead carp *Hypophthalmichthys nobilis* and silver carp *Hypophthalmichthys molitrix*. PLoS One. 2013;8(8):e73829. doi: 10.1371/journal.pone.0073829 23967350 PMC3743794

[3] Kolar CS, Chapman DC, Courtenay WR Jr, Housel CM, Williams JD, Jennings DP, et al. Bigheaded carps: a biological synopsis and environmental risk assessment, Vol. 33. American Fisheries Society Special Publication; 2007. p. 1–204. https://pubs.usgs.gov/publication/70161142

[4] Nico LG, Williams JD, Jelks HL. Black carp: biological synopsis and risk assessment of an introduced fish, Vol. 32. American Fisheries Society Special Publication; 2005. p. 1–337. doi: 10.1899/27.3.BR.800.1

[5] Garcia T, Zuniga Zamalloa C, Jackson PR, Murphy EA, Garcia MH. A laboratory investigation of the suspension, transport, and settling of silver carp eggs using synthetic surrogates. PLoS One. 2015;10(12):e0145775. doi: 10.1371/journal.pone.0145775 26713855 PMC4699218

[6] Guo H, Li Y, Yang W, Chen D, Huang M, Xing L. Settling and transport properties of grass carp and silver carp eggs in the water-hardened phase: implications for resource protection and invasion control during early life period. Ecol Indicators. 2023;148:110064. doi: 10.1016/j.ecolind.2023.110064

[7] Velandia RA, Campo-Nieto O, Márquez EJ. Astyanax caucanus: microsatellite loci development and population genetics in the Cauca River, Colombia. Hydrobiologia. 2024;851(8):2007–24. doi: 10.1007/s10750-023-05434-w

[8] Yih P, Liang T. Natural conditions of the spawning grounds of the domestic fishes in yangtze river and essential external factor for spawning. Acta Hydrobiol Sin. 1964;5(1):1–15. doi: 10.3724/issn1000-3207-1964-1-1-f

[9] Chen Q, Zhang J, Chen Y, Mo K, Wang J, Tang L, et al. Inducing flow velocities to manage fish reproduction in regulated rivers. Engineering. 2021;7(2):178–86. doi: 10.1016/j.eng.2020.06.013

[10] Prada AF, George AE, Stahlschmidt BH, Chapman DC, Tinoco RO. Survival and drifting patterns of grass carp eggs and larvae in response to interactions with flow and sediment in a laboratory flume. PLoS One. 2018;13(12):e0208326. doi: 10.1371/journal.pone.0208326 30566492 PMC6300213

[11] Vasconcelos LP, Alves DC, Agostinho AA, Baumgartner LJ, Pelicice FM, Lima FP, et al. Fish eggs and larvae drifting through hydropower reservoirs: a case study in the Brazilian Amazon. Hydrobiologia. 2022;849(2):357–72. doi: 10.1007/s10750-021-04631-9

[12] Prada AF, George AE, Stahlschmidt BH, Jackson PR, Chapman DC, Tinoco RO. Using turbulence to identify preferential areas for grass carp (*Ctenopharyngodon idella*) larvae in streams: a laboratory study. Water Resour Res. 2021;57(2):e2020WR028102. doi: 10.1029/2020wr028102

[13] Liang ZX, Yi BL, Yu ZT. The reproductive habit and embryonic development of elopichthys bambusa in changjiang (Yangtze River)[J]. Acta Hydrobiologica Sinica. 1984;8(4):389–403.

[14] Chapman DC, George AE. Developmental rate and behavior of early life stages of bighead carp and silver carp. Sci Investig Rep. 2011;5076:1–62. doi: 10.3133/sir20115076

[15] Alvarez LV, Schmeeckle MW, Grams PE. A detached eddy simulation model for the study of lateral separation zones along a large canyon‐bound river. JGR Earth Surf. 2017;122(1):25–49. doi: 10.1002/2016jf003895

[16] Beechie TJ, Liermann M, Pollock MM, Baker S, Davies J. Channel pattern and river-floodplain dynamics in forested mountain river systems. Geomorphology. 2006;78(1–2):124–41. doi: 10.1016/j.geomorph.2006.01.030

[17] Constantinescu G, Miyawaki S, Rhoads B, Sukhodolov A, Kirkil G. Structure of turbulent flow at a river confluence with momentum and velocity ratios close to 1: Insight provided by an eddy-resolving numerical simulation. Water Resour Res. 2011;47(5):W05507. doi: 10.1029/2010wr010018

[18] Li G, Elliott CM, Call BC, Sansom BJ, Jacobson RB, Wang B. Turbulence near a sandbar island in the lower Missouri River. River Res Appl. 2023;39(12):1857–74. doi: 10.1002/rra.4180

[19] Haworth MR, Bestgen KR, Butler SE, Combs DM, Hardy RS, Hines BA, et al. Flow and water temperature affect reproduction and recruitment of a Great Plains cyprinid. Can J Fish Aquatic Sci. 2016;74(6):853–63. doi: 10.1139/cjfas-2016-0238

[20] George AE, Garcia T, Chapman DC. Comparison of size, terminal fall velocity, and density of bighead carp, silver carp, and grass carp eggs for use in drift modeling. Trans Am Fish Soc. 2017;146(5):834–43. doi: 10.1080/00028487.2017.1310136

[21] Prada AF, George AE, Stahlschmidt BH, Jackson PR, Chapman DC, Tinoco RO. Influence of turbulence and in-stream structures on the transport and survival of grass carp eggs and larvae at various developmental stages. Aquatic Sciences. 2019;82(1):16. doi: 10.1007/s00027-019-0689-1

