# Expression of Concern: From spawn to survival: decoding the hydraulic conditions for successful silver carp egg incubation

**DOI:** 10.1371/journal.pone.0358617

**Published:** 2026-09-21

**Authors:** The PLOS One Editors

## Notice of Republication

Following the publication of this article [1], concerns were raised regarding incorrect information in the reference list of the published article. Specifically:

- Four of the cited references could not be verified by PLOS as consistent with any articles in the published literature.
- A further five references contained errors such as an incorrect DOI and/or author list,
- Several articles cited did not appear to support the corresponding statements associated with the in-text citation.

Additionally, the *PLOS One* Editors identified concerns about the article’s peer review.

We regret that the issues were not identified prior to the article’s publication.

Editorial follow-up noted that the unverified reference errors were not present in the References list of the initial submission and were introduced during revision of the manuscript. The corresponding author stated that those errors arose when the manuscript underwent revisions by multiple contributors using differing reference management software, and they provided a corrected reference list.

In light of the above issues, the *PLOS One* Editors issue this Expression of Concern. Readers are advised to interpret the article [1] with caution.

The article [1] was republished on August 26, 2026, to address the errors in the references and to replace the references that could not be verified. Additionally, a typesetting error in the text of the Materials and Methods section, subsection 2.3 Characterization of flow conditions, where symbols representing the variables were not published correctly, has been corrected.

Please download this article again to view the correct version. The originally published, uncorrected article and the republished, corrected article are provided here for reference.

The publisher apologizes for the typesetting error.

## Supporting information

S1 File Originally published, uncorrected article. (PDF)

S2 File Republished, corrected article. (PDF)
