# Supplementary material for: From spawn to survival: decoding the hydraulic conditions for successful silver carp egg incubation
Source: PLoS One. 2025 Apr 22;20(4):e0320798. doi: 10.1371/journal.pone.0320798 (PMC12013886; doi:10.1371/journal.pone.0320798)
Supplement: S1 Fig — (DOCX) [file pone.0320798.s001.docx]

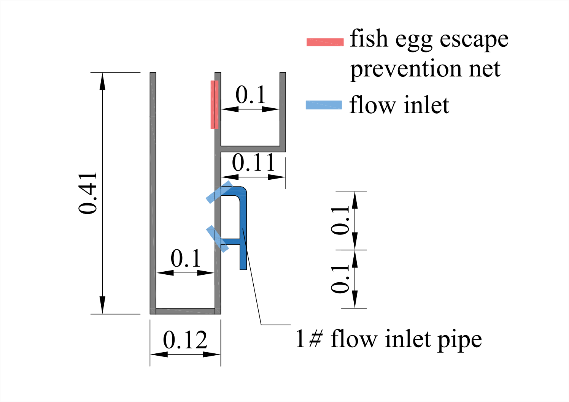


**S1 Fig.** Longitudinal section and dimension annotation of incubation channel and buffer channel, and the inflows setup.
