# Supplementary material for: From spawn to survival: decoding the hydraulic conditions for successful silver carp egg incubation
Source: PLoS One. 2025 Apr 22;20(4):e0320798. doi: 10.1371/journal.pone.0320798 (PMC12013886; doi:10.1371/journal.pone.0320798)
Supplement: S2 Fig — (DOCX) [file pone.0320798.s002.docx]

| 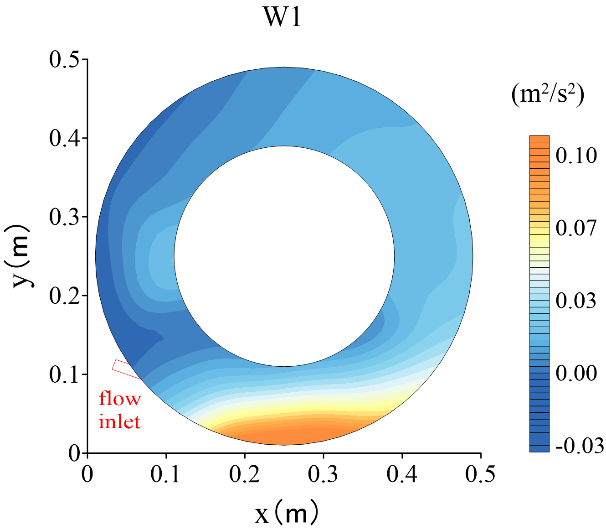  (a) Average turbulence energy 0.0119m^2^/s^2^ | 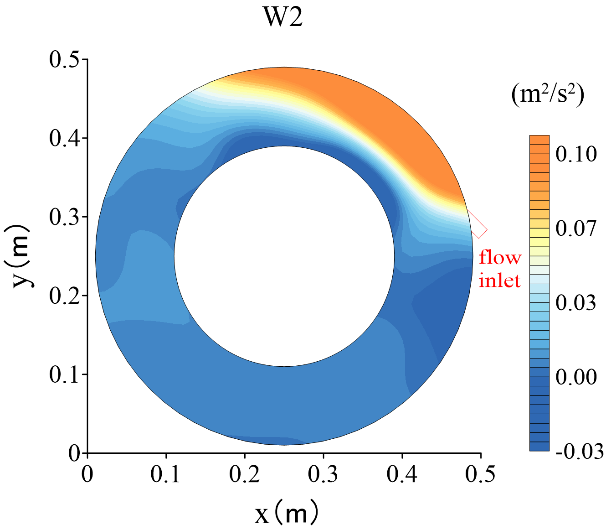  (b) Average turbulence energy 0.0166m^2^/s^2^ |
| --- | --- |
| 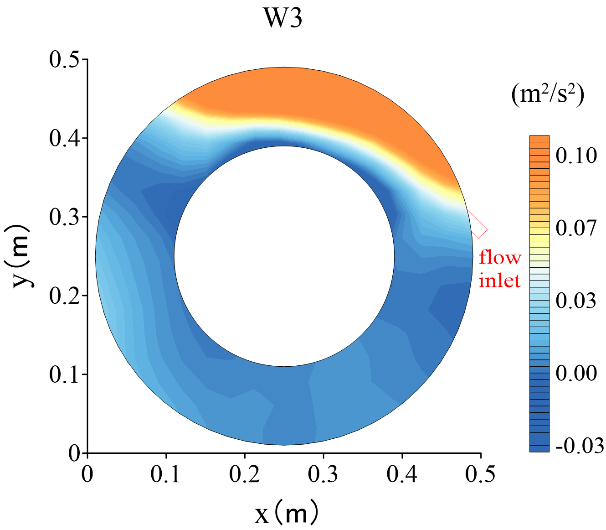  (c) Average turbulence energy 0.0249m^2^/s^2^ | 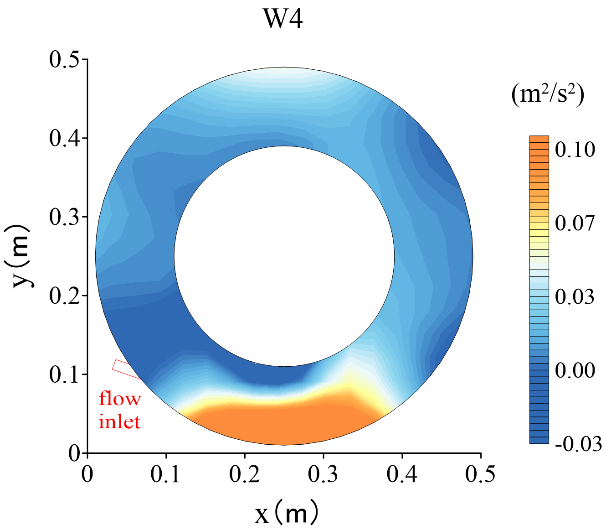  (d) Average turbulence energy0.0281m^2^/s^2^ |

**S2 Fig.** Turbulent Energy Distribution in Incubation Channel at Constant Flow Velocity (0.7 m/s).
