# Supplementary material for: From spawn to survival: decoding the hydraulic conditions for successful silver carp egg incubation
Source: PLoS One. 2025 Apr 22;20(4):e0320798. doi: 10.1371/journal.pone.0320798 (PMC12013886; doi:10.1371/journal.pone.0320798)
Supplement: S1 File — (DOCX) [file pone.0320798.s003.docx]

**S1 File** Introduction of ADV

The ADV probe, comprising one transmitting sensor and four receiving sensors, was configured using Vectrino-II software. We set a 25 Hz sampling frequency with 100s sampling time per point. The software provided real-time data on velocity (*v*), signal-to-noise ratio (SNR), signal strength (Amp), and correlation coefficient (Corr).

Data quality was unsured by selecting measurements with correlation coefficients above 80% and SNR exceeding 15db is generally required. When necessary, PIV tracer particles were added to improve SNR. Post-processing involved converting NTK files to mat format and analyzing with MATLAB to calculate average velocity and turbulence intensity for each observation point.
